# NMR reaction monitoring in flow synthesis

**DOI:** 10.3762/bjoc.13.31

**Published:** 2017-02-14

**Authors:** M Victoria Gomez, Antonio de la Hoz

**Affiliations:** 1 Área Química Orgánica, Facultad de Químicas, Universidad de Castilla-La Mancha, Avda. Camilo José Cela nº 10, E-13071 Ciudad Real, Spain and Instituto Regional de Investigación Científica Aplicada (IRICA), Avda. Camilo José Cela s/n, E-13071 Ciudad Real, Spain

**Keywords:** expert systems, flow chemistry, microcoil, NMR probes

## Abstract

Recent advances in the use of flow chemistry with in-line and on-line analysis by NMR are presented. The use of macro- and microreactors, coupled with standard and custom made NMR probes involving microcoils, incorporated into high resolution and benchtop NMR instruments is reviewed. Some recent selected applications have been collected, including synthetic applications, the determination of the kinetic and thermodynamic parameters and reaction optimization, even in single experiments and on the μL scale. Finally, software that allows automatic reaction monitoring and optimization is discussed.

## Introduction

New enabling technologies have facilitated the transition from traditional chemistry to a more automated approach that will be the chemistry of the 21^st^ century [1–2]. The objective is that the reaction, analysis and work-up can be performed in an automatic and continuous manner, but optimization and scale-up represent a new step forward towards the full automation of the chemical process [3]. The final objective is to save time for chemists to focus on the more technical work and to spend their time planning, interpreting results and developing new projects.

In this regard, flow chemistry is the central motif of this automated approach. In contrast to batch mode, in flow chemistry the starting materials are continuously introduced into the flow reactor (e.g., a microreactor or a column) and the product is continuously eluted from the end of the flow reactor. This approach can be used from microscale to laboratory scale and even to production scale [4–5].

Some important advantages of flow chemistry are:

- Diffusion is clearly improved with regard to chemistry in batch (reagents and products), thus leading to improved heat and mass transfer.
- The surface to volume ratio increases with regard to reactions in batch. This enables good control of the reaction temperature and resolves the problems of highly exothermic reactions.
- Dangerous or air- and moisture-sensitive compounds can be used safely due to the small amounts of reagents and the use of a closed system with efficient control of pressure.
- The use of solvents can be minimized since concentrations can be increased up to the limit of solubility.
- Coupling with other enabling technologies is very simple and more efficient than in batch (photochemistry, electrochemistry, microwave, ultrasound, etc.) [6–9].

In this regard, in a recent paper a compact reconfigurable flow system was described for the continuous flow production of pharmaceuticals. The system comprised different types of preparation, reaction and elaboration modules that could be coupled in different configurations and the authors used them to prepare from hundreds to thousands of doses of pharmaceuticals that fulfilled the quality standards of the pharmacopeia [10].

In research laboratories that focus on rapid, reproducible and efficient analysis and optimization, and on the production scale for quality control, the coupling of flow and microreactor technology with a good analytical method is a prerequisite. Several analytical methods have been used and these include fluorescence, ultraviolet–visible (UV–vis), RAMAN, infrared (IR) and nuclear magnetic resonance (NMR) spectroscopy and mass spectrometry (MS). The use of a particular technique depends on the application, on the characteristics of the analyte and the ease of coupling with the flow system [11–12].

In this paper we focus on the coupling of nuclear magnetic resonance spectroscopy with flow and microreactor systems for the rapid analysis and optimization of reaction parameters and conditions. The use of this technique in mechanistic studies is also discussed.

## Review

### Commercial flow probes

NMR spectroscopy is based on the absorption of radiofrequency radiation to produce absorption on the nuclear spin level when nuclei are submitted to a strong magnetic field [13]. NMR spectroscopy is one of the most powerful and versatile methods for structural determination, enabling qualitative and quantitative analysis of samples. It can be applied to almost all elements in the periodic table, the only requirement being the presence of an isotope, not necessarily the most abundant, that shows magnetic properties.

The main drawback of NMR spectroscopy is that the sensitivity is very low when compared with other spectroscopic techniques such as UV, since the difference in population between the ground and the excited state is very low and is strongly dependent on the permanent magnetic field (*B*_0_) applied. This limitation is compensated by using stronger magnetic fields, which results in more complex, large and expensive NMR instruments and/or the development of specialized probes. Although the low sensitivity of NMR spectroscopy is a disadvantage for an analytical method, the power of this technique in structural determination compensates for its limitations.

The application of NMR spectroscopy to analytical chemistry in flow, preparative flow chemistry and microreactor technology requires the use of specially designed equipment, especially flow probes, flow cells or specialized microfluidic coils. In most cases, high-resolution NMR instruments are used but the high cost of these systems and the large space required limit their application on the laboratory scale and lab-on-a-chip. Recently, benchtop low field NMR instruments have been introduced in flow chemistry to overcome these limitations, with the advantage of lower cost and better integration with the continuous flow platform since the whole system can be set up in a fume hood. The main drawback of these systems is the lower resolution and sensitivity as compared to high resolution NMR instruments, which limits the application of benchtop NMR instruments to relatively simple structures.

**Figure 1:**
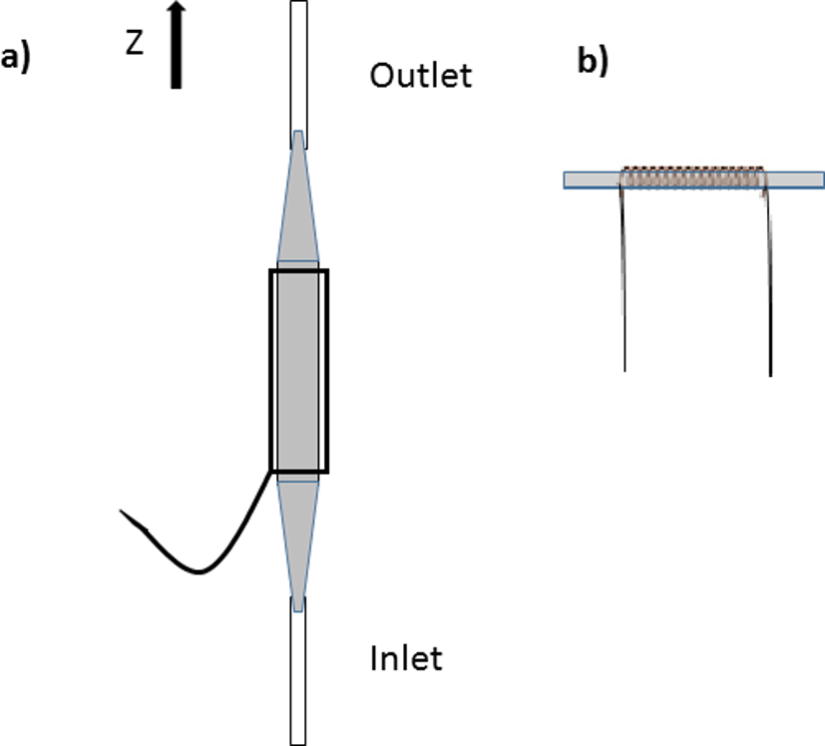
Graphical representation of (a) conventional flow cell with a saddle-shaped RF coil and (b) flow capillary with a solenoid coil.

Two classes of flow probes have been designed depending on the position of the sample tube, which can be vertical (denoted below as type 1) or horizontal (denoted below as type 2) (Figure 1), and the shape of the RF coil for transmitting and receiving, namely saddle-shaped when the sample tube is placed vertically and solenoidal when the sample tube is placed horizontally [14]. Manufacturers have designed commercial NMR flow probes of type 1 that can be integrated into their high and low-resolution NMR instruments. Type 2 probes have been designed by different research groups and integrated into standard NMR instruments.

Flow probes can further be classified as ‘room temperature probes’ if the RF coils and the sample are at similar temperatures or as ‘cryogenic probes’ if the RF coils are insulated from the sample chamber and kept cold.

The development of commercial NMR flow probes requires a different design when compared to the standard tube-based probes. For the design of a NMR flow probe, similarly to the design of a new probe head, careful choice of the components should be made in order to have an optimal resolution, sensitivity and RF homogeneity, but in addition, other factors should be taken into account because of working on-flow. Hence, the design should allow a high filling factor, the flash out of air bubbles and the displacement of the existing fluid in the detection volume by the incoming fluid instead of just mix with it, among other issues [14]. Hence, the flow cell is a sample tube (made of glass or quartz) with openings at the top (outlet) and at the bottom (inlet) to enable connection to the flow system. NMR flow cells generally have a larger inner diameter at the centre than at the ends (Figure 1). The larger central portion of the flow cell is the sample chamber, i.e., the detection zone. The smaller segments at the ends correspond to the inlet and the outlet stems. When designing a flow cell, the total volume of the sample chamber is considered to be the minimum volume and this must be twice the active volume. This ratio is similar to that found for 5 mm NMR tubes in standard probes.

Flow cells are usually made of quartz, although Pyrex^®^, sapphire and alumina can also be considered. Quartz is the material of choice because of its uniformity, purity and mechanical strength. Moreover, quartz is a machinable material and shows excellent electrical properties. Tubing connections are made of PEEK (poly-ether-ketone) due to the strength of this material. However, PEEK has three main drawbacks, it absorbs DMSO and CH_3_OH, it is not compatible with acids and it does not have a good turn radius.

Finally, most standard flow probes include pulsed-field gradient hardware, which enables interesting uses for the probe such as gradient shimming, solvent suppression pulse sequences (i.e., WET, which is especially suitable for applications in flow), and the use of pulse sequences that incorporate gradients, nowadays commonly found within most NMR experiments.

Considering the points outlined above, several advantages of flow NMR over traditional NMR can be envisaged. Firstly, additional time is not required to lock or shim each sample when the solvent is kept constant during the experiment. Secondly, deuterated solvents are not required because of WET solvent suppression and also because locking is not required. Thirdly, more samples can be analyzed automatically from microtiter plates, thus avoiding the use and possible breakage of glass sample tubes.

### Microcoil probes

An interesting way to increase the sensitivity of NMR is the use of microcoil probes [15–16]. Based on the reciprocity principle [15], it has been shown that for a constant length-to-diameter ratio, the NMR detector (i.e., coil) sensitivity is inversely proportional to its diameter. For a volume-limited sample, the signal is maximized when the coil is scale-down to enclose this volume sample. Although these probes show several advantages, as for instance are the coupling into continuous flow systems and its integration in compact magnets due to their lower requirements for the spatial *B*_0_ field homogeneity, the construction of the probe for the highest sensitivity and resolution is a challenging task. The latter falls outside the scope of this review and instead, we will describe the types and features of microcoil probes in this section and its integration with flow systems in the following sections.

**Figure 2:**
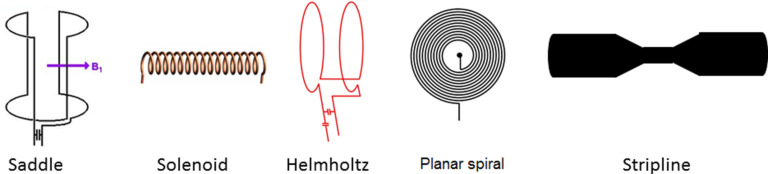
Possible geometries of NMR coils.

An important requirement in NMR spectroscopy is that a sufficiently strong *B*_1_ is generated perpendicular to the static *B*_o_ field. The geometry of the coil is very important in order to generate a homogeneous *B*_1_ field over the entire sample volume. Hence, the geometry of the coil should be optimized in order to obtain the highest possible sensitivity and resolution. The most widely used geometries for NMR coils are represented in Figure 2. The most typical geometry used in commercial solution NMR probes is the saddle type. Although this geometry generates a very homogeneous magnetic field orthogonal to the permanent field *B*_0_, it is not suitable for miniaturization. As a consequence, the saddle coil it is not used in small-volume NMR applications.

The main geometries reported for microcoils are, solenoidal, flat helical (also called planar microcoils), microslot and stripline (Figure 2). Below, the planar and solenoid coils are discussed more in detail as they are the most reported in literature.

### Microsolenoid coils

A coil of helical geometry is wrapped around a capillary adopting the size and shape of the sample and therefore, a good filling factor is achieved. Solenoid coils have been investigated in detail. A representative example was reported by Sweedler et al. [17] who designed a microsolenoid coil with a detection cell volume of ca. 5 nL, and line widths of only 0.6 Hz for a neat ethylbenzene sample. Recently, a new manufacturing procedure, by using a sacrificial layer and a combination of solvents, has been reported by Gruschke et al. [18] yielding a maximized and optimum filling factor compared to former procedures as hollow microcoils are encased in external support structures. A new methodology for the easy fabrication of solenoid coils has been reported by Saggiomo and Velders [19] using 3D-printing technology. The authors described an easy two-step ABS (acrylonitrile butadiene styrene) scaffold removal method to obtain a 3D printed device inserted in a block of PDMS (polydimethylsiloxane). The authors tested this methodology for the creation of microfluidic devices but they also fabricated a simple, cheap and sensitive NMR microsolenoid [19] with a detection volume of only 2 μL. Integration with a 9.4 T superconducting magnet allowed them to obtain high-resolution NMR spectra.

The main advantages of microsolenoid coils are: Excellent *B*_1_-field uniformity and *B*_1_/i field efficiency resulting in a high signal-to-noise ratio (SNR). In comparison to planar coils, the solenoid have lower resistance, better nutation curves (reaching the ideal sinusoidal behaviour) representing a much more uniform *B*_1_ field than planar coils, however solenoid coils show lower resolution than planar coils in a comparative study reported by Popovic et al. [20] when both types of coils were fabricated following the same process.

And the main disadvantages of microsolenoid coils: Tedious manufacturing procedure especially for very small volumes as well as encountering the optimum position of the sample in the coil. Solenoid coils are usually wound by hand, resulting in a low reproducible and very time-consuming process.

### Planar coils

Spiral planar coils were introduced in NMR spectroscopy derived from the semi-conductor industry by means of microfabrication techniques. Planar coils were studied in detail by Popovic et al. [21] as an alternative to solenoid coils. Planar coils show the following advantages:

They can be batch-fabricated with submicrometer resolution and with a high degree of geometric precision and reproducibility by standard photolithographic techniques. In addition, they can be integrated with chip-based microfluidic systems. To end with the advantages, planar coil facilitate an increased throughput since an array of planar coils and microfluidic channels can be manufactured by microfabrication techniques [15–16,21].

The disadvantages of planar coils are: Planar coils suffer of a high series resistance resulting in a low SNR as the latter is dominated by the thermal noise of the coil. The SNR depends on the geometrical features of the coil. For instance, the number of turns is crucial since a large number of turns can increase the unitary field produced by the coil but will also lead to higher resistance [21]. It is believed that the nearby windings of the coil induce static field distortions resulting in lower resolution and sensitivity in the NMR spectrum [22]. The optimum dimensions for a planar microcoil were presented by van den Berg et al. [23] and obtained from finite-element simulations [24]. High SNR were obtained at a low-field magnet. Another disadvantage of planar coils is the weak and inhomogenous *B*_1_-field produced by the coil resulting in a non-sinusoidal nutation curve and in low SNR of the free induction decay [20].

Despite these disadvantages, interesting applications of planar microcoils can be found in literature. Hence, Velders et al. [25] studied supramolecular interactions by ^19^F NMR spectroscopy at the picomole level. This application takes advantage of the high sensitivity and large chemical-shift dispersion of this nucleus. The authors determined the association constant of the complex of NaPF_6_ with α-cyclodextrin at the picomole level with a detection volume of 50 nL and using non-deuterated solvents [25].

To conclude with the different coil geometries, microslot NMR microprobes and stripline coils show also interesting applications in small-volume NMR spectroscopy. A microslot consists of a dual-layer metallic microstrip that can have submicrometer dimensions. These coils produce field lines that are more homogeneous than those obtained with planar coils or just a metallic wire and find applications even for NMR metabolomics [26]. Stripline coils represent a simple and effective coil design with interesting applications even as detectors in DNP methods [22,27]. Stripline coils produce high and homogeneous *B*_1_ field, can be integrated on a microfluidic chip and show scalability as reported by Kentgens et al. [22].

### Applications of flow NMR in reaction monitoring

Keifer defined flow-NMR [28] as any NMR technique in which the sample flows through a tube into the NMR probe at some time during the measurement process.

The first reported use of a flow-NMR technique was in 1951 [29], when the ^1^H spectrum of water (doped with FeCl_3_) was recorded as it flowed through the NMR probe. In the seventies, a group of related techniques that were variously called ‘stopped-flow NMR’ [30], ‘rapid-injection NMR’ [31], ‘continuous-flow’ NMR [32], or just ‘flow NMR’ [33] were introduced and their use has continued to the present day. All of these techniques involve the use of standard NMR probes and do not require specialized equipment.

The introduction of LC–NMR was a natural development, although LC–NMR requires the use of an NMR probe that is dedicated solely to the observation of a sample flowing through tubing from another source. This requirement led to the investigation, design and development of the NMR flow probe. Several techniques were developed for the integration of the two systems, such as on-flow and stopped-flow LC–NMR. In the on-flow technique the solvent stream flows continuously during the analysis. However, one important problem that must be addressed is to achieve a good NMR signal-to-noise ratio. This limitation is more important than the chromatographic resolution. The NMR signal-to-noise ratio can be improved by signal averaging, but this approach usually requires the flow to be stopped for substantial periods of time.

The terms in-line and on-line analysis have been commonly used. ‘In-line’ and ‘on-line’ refer to methods of analysis that do not require the manual transfer of samples [34]. When the NMR probe and the reaction system are connected in-series, all of the reaction mixture passes through the NMR instrument and is continuously analyzed. This method is called in-line analysis. This configuration minimises the time-lag between reaction and analysis. For on-line analysis the NMR system is not directly connected to the reaction system and the sample is transferred from the reaction to the analysis system with representative aliquots collected periodically during the reaction. This method is simpler and can be used when direct connection is difficult to be analyzed by NMR. A similar definition is provided by the FDA: “on-line: Measurement where the sample is diverted from the manufacturing process, and may be returned to the process stream. In-line: Measurement where the sample is not removed from the process stream and can be invasive or non-invasive” [35].

**Figure 3:**
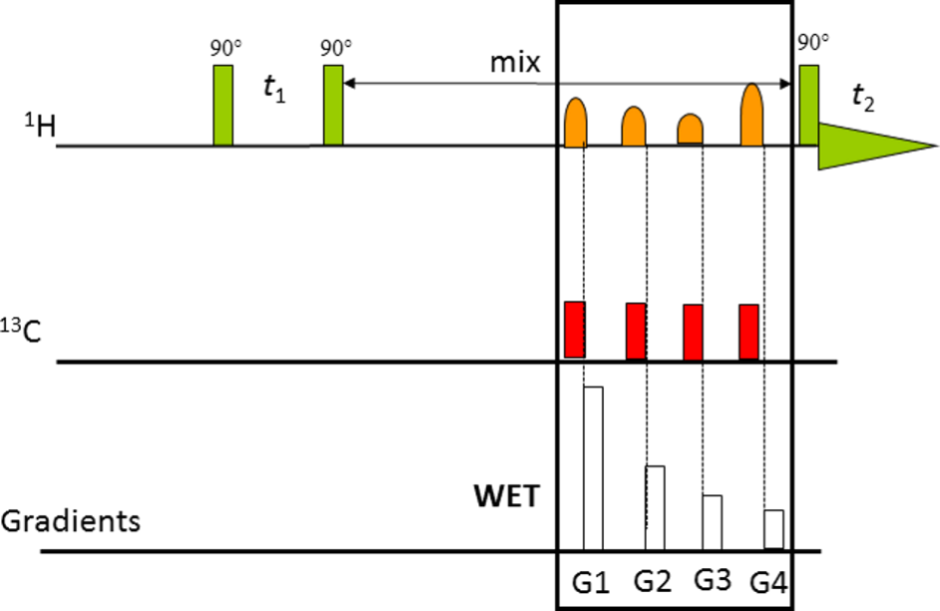
The NMR pulse sequence used for NOESY with WET solvent suppression [28].

If the solvent contains protons, solvent suppression pulse sequences have to be used to obtain a good quality NMR spectrum. The first sequences used were presaturation and binomial sequences. The introduction of the WET sequence for solvent suppression was an important advance. WET has several advantages in that solvent suppression is fast, so it works well with flowing samples, it can supress multiple solvent lines and it is more frequency selective than other techniques. In contrast, pre-saturation do not work well in flowing samples and it is slower in its recycle rate. WET has also been incorporated into all of the standard 2D NMR sequences [28]. As an example Figure 3 shows a NOESY pulse sequence in which the WET sequence is incorporated into the end of the mix delay.

NMR spectroscopy can be coupled to most separation techniques, including gas chromatography (GC), supercritical fluid chromatography (SFC), gel-permeation chromatography (GPC), high-performance liquid chromatography (HPLC), capillary electrophoresis (CE), capillary LC–NMR (CapLC–NMR), capillary electrochromatography-NMR (CEC–NMR), capillary isotachophoresis (cITP) and size-exclusion chromatography-NMR (SEC–NMR) [28].

Hyphenation is another important field in which separation and analytical techniques are combined. Hyphenation involves adding on other analytical techniques, almost as if they were ‘building blocks’, for instance, LC–NMR–MS, which was first described in 1995.

Flow Injection Analysis-NMR (FIA–NMR) and Direct Injection-NMR (DI–NMR) were the first non-chromatographic flow-NMR methodologies to be introduced. By simply removing the chromatography column LC–NMR produces FIA–NMR, a technique that has the capability of performing multiple analyses rapidly.

In contrast to FIA–NMR, DI–NMR lacks a mobile phase, just the solvent to dissolve the sample and some additional to rinse the flow cell. Also, the pump is simplified and the sample is injected directly into the flow probe to give a simple flow-NMR system. Applications of DI–NMR include combinatorial chemistry for the analysis of libraries [36], analysis of biofluids for clinical diagnosis [37] and metabolomics [38].

### Applications in organic synthesis

In this section we will discuss some recent selected examples of the application of NMR reaction monitoring in flow chemistry. These examples include the design of flow systems, the use of standard NMR instruments and flow probes, the use of microcoils and finally the use of flow-NMR for kinetic and mechanistic studies and for the optimization of synthetic processes.

**Figure 4:**
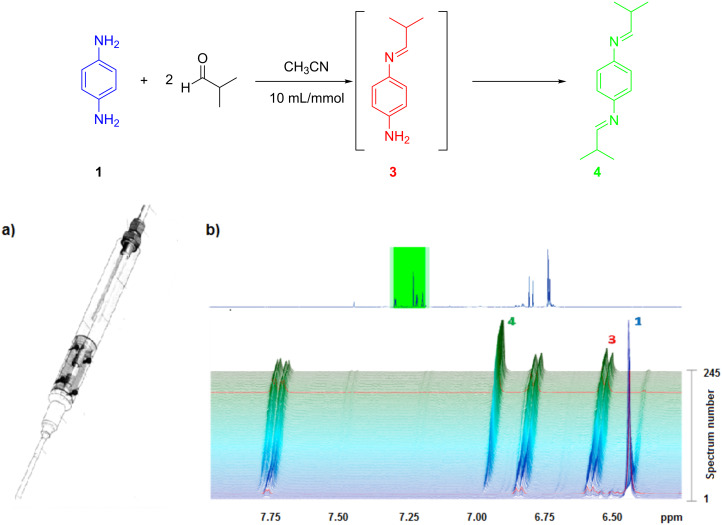
Reaction of *p*-phenylenediamine with isobutyraldehyde. (a) Flow tube and (b) ^1^H NMR stacked plot (400 MHz). NMR signals used to monitor the reaction (245 spectra were recorded in 47 h). Reproduced with permission from reference [39]. Copyright 2014 The American Chemical Society.

Marquez et al. [39] developed a new NMR flow tube for the use in a standard 5 mm NMR probe (Figure 4). This system allows experiments to be carried out on flowing samples. The authors tested this flow tube to monitor the standard reaction of *p*-phenylenediamine and isobutyraldehyde to form the diimine product and good results and reproducibility were obtained.

The authors consider that this technology can be used to determine the mechanistic and kinetic aspects of reactions without a specialized flow probe and using different kinds of spectrometers with varying magnetic field strengths.

Danielli et al. [40] described the application of Benchtop NMR spectroscopy in flow reactions (SpinSolve from Magritek at 60 MHz). They considered that the field homogeneity and sensitivity that compact NMR spectrometers provide is sufficient to analyze small molecules at concentrations of 1 mmol L^−1^ in single-scan experiments. As a proof-of-concept, they studied the transfer hydrogenation process of acetophenone with isopropanol catalysed by iridium complexes. The reaction was performed in batch and the sample was introduced into the magnet with a pump and Teflon tubing to form a closed circuit, at a flow rate of 1 mL min^−1^. The kinetic rate could be studied as a function of the catalyst concentration and good agreement was found with the results obtained by gas chromatography. As expected for a first-order reaction, a linear dependence of the kinetic constant on the catalyst concentration was found.

**Figure 5:**
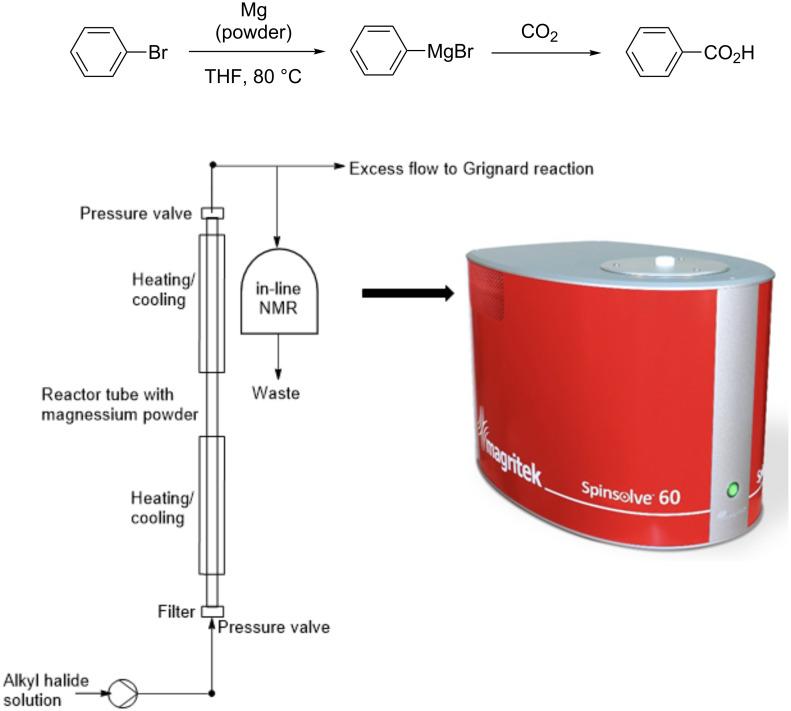
Scheme and experimental setup of the flow system.

An interesting point to consider is the comparison of in-line and off-line analysis. For example, Duchateau et al. [41] described the preparation of Grignard reagents from aryl halides and magnesium using a fluidized bed reactor under continuous-flow conditions. In a second flow reactor the Grignard was reacted with CO_2_ to obtain carboxylic acids (Figure 5). The whole process was monitored by on-line ^1^H NMR spectroscopy using a low field NMR instrument (Spinsolve-60 from Magritek).

The reaction was analysed by in-line NMR and off-line with a standard NMR tube. In the first case, the amount of oxidized Grignard reagent was significantly lower, showing the advantages of in-line measurements. In the in-line experiment the reaction mixture was introduced into the flow NMR cell at 1 mL min^−1^ showing a conversion of about 80% in 70 min.

In this regard, Foley et al. [42] reported a comparison of three different methods for the analysis of flow reactions: online NMR, static NMR tubes, and periodic inversion of NMR tubes, using a high-resolution NMR instrument (400 MHz). Both studied reactions, heterogeneous reactions with long reaction times and homogeneous reactions with short reaction times showed that mixing has an important effect on the final result.

A careful evaluation of the three analytical methods and reaction conditions showed that the NMR technique has a significant influence on the results of the analysis. Considering the application of interest, the choice of one or other method could be crucial. In this regard, flow NMR gives more accurate results for kinetic studies, while static NMR is suitable to obtain structural information and determination of the mechanism.

The NMR instrument should also be evaluated considering that high-resolution NMR instruments are expensive, in terms of both acquisition and maintenance, and they require special laboratory installation. Benchtop NMR instruments have low cost and low maintenance; they can be easily placed in a conventional laboratory fume hood and transported to the required place.

Elipe and Milburn [43] studied the pros and cons of a benchtop NMR instrument at 45 MHz (Pico Spin-45). For this purpose, they studied reactions like the Fisher esterification, Suzuki reactions, and oxime formation and they analyzed the samples by simple injection of aliquots in the inlet port through an HPLC filter using non-deuterated solvents.

The major advantages of low field NMR arise from its simplicity, especially the fact that they do not require cryogenic liquids for the magnet, and they have reduced maintenance costs and also simpler handling and operation. Although there is a clear reduction in the sensitivity with regard to high field instruments, it is possible to obtain a ratio of up to 10:1 and this is sufficient for many applications. The major problems arise in reactions involving complex structures with small chemical shift dispersion and second-order coupling, which produce complex spectra with several overlapping signals. Finally, many low field instruments are supplied without variable temperature units and this limits the application to reactions at room temperature or close to the temperature of the magnet (25–50 °C, 42 °C in this case).

Another issue that must be considered when using benchtop NMR instruments is the use of magnetic stirrers, which can generate fluctuating magnetic fields that interfere with the NMR measurement if they are close to the magnet in the fume hood [40]. Consequently, it is advisable to use mechanical stirrers for such reactions.

As pointed out above, the use of microcoils increases the sensitivity in NMR analysis. Moreover, it is possible to use these in conjunction with microreactors and consequently to design integrated systems that can be classified in the lab-on-a-chip methodology.

**Figure 6:**
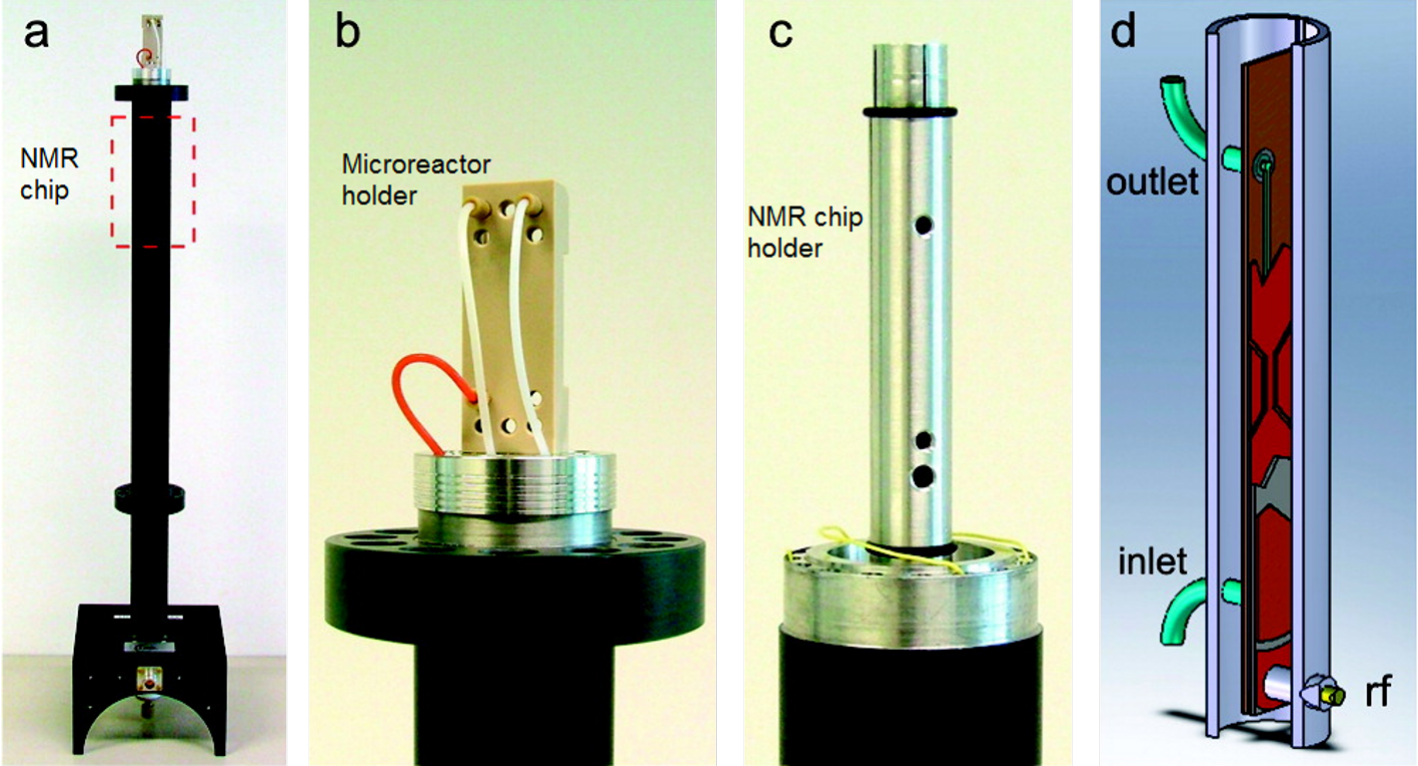
(a) Microfluidic probe. (b) Microreactor holder. (c) Stripline NMR chip holder. (d) Arrangement of the microfluidic chip in the holder. Reproduced with permission from reference [44]. Copyright 2009 The American Chemical Society.

**Figure 7:**
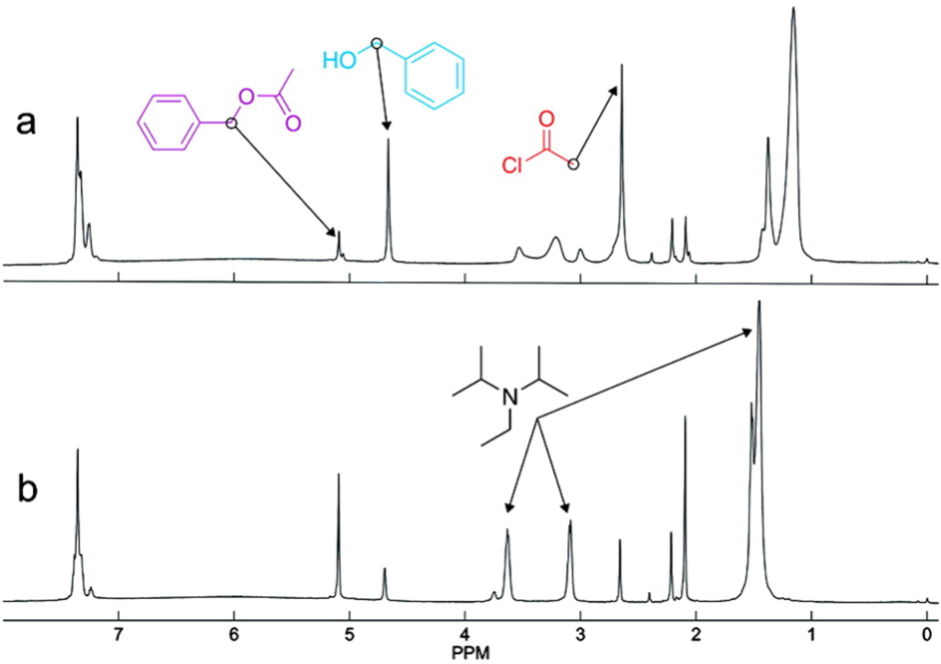
Acetylation of benzyl alcohol. Spectra at (a) 9 s and (b) 3 min. Stoichiometry: benzyl alcohol/DIPEA/acetyl chloride 1:1:1.2. Reproduced with permission from reference [44]. Copyright 2009 The American Chemical Society.

An interesting example was developed by Kentgens et al. [44], who designed a stripline microcoil for NMR studies coupled to a microreactor (Figure 6). The system was coupled to a custom-made NMR probe and inserted into a high-resolution NMR instrument (600 MHz). As pointed out above, the authors demonstrated that stripline microcoils show higher sensitivity than solenoid and planar microcoils with a line of <1 Hz in ethanol. As a proof-of-concept, the integrated flow system (microreactor-stripline NMR chip) was tested in the acylation of benzyl alcohol with acetyl chloride (Figure 7) using DIPEA as the base. The kinetics were studied by in situ monitoring and it was found that 70% conversion was achieved after 3 minutes. Broadening observed in the DIPEA signals is a consequence of protonation.

This example clearly shows that it is possible to integrate in one compact system the microreactor and the NMR chip to analyze raw samples and to apply this system to monitor reactions in a lab-on-a-chip approach.

### Kinetic and mechanistic studies

The rapid analysis produced in flow NMR can be used for the detection of reactive intermediates and consequently for studying reaction mechanisms and the rapid optimization of a chemical process.

**Figure 8:**
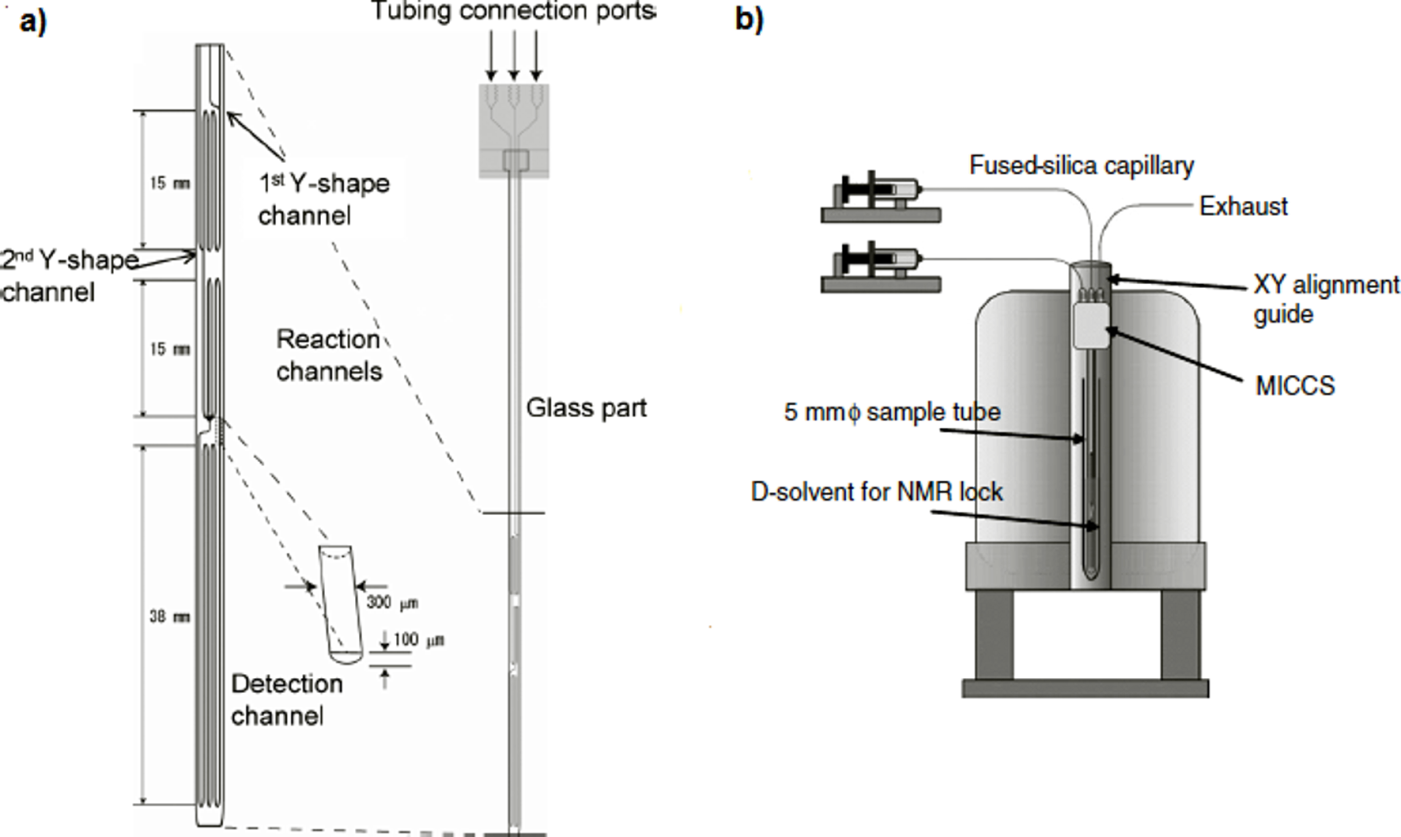
a) Design of MICCS and b) schematic diagram of MICCS–NMR [45]. CH_2_Cl_2_ solutions of oxime ether and triethylborane were introduced into MICCS by two inlet ports and mixed at the first Y-shape channel (Flow rate 5 μL min^−1^). A CH_2_Cl_2_ solution of methanol was introduced and mixed with reaction mixture at the second Y-shape channel to quench the reaction. Reproduced with permission from reference [45]. Copyright 2007 John Wiley and Sons.

The first example was described by Nakakoshi et al. [45], who developed a micro-channelled cell for synthesis and monitoring (MICCS) (Figure 8) and this was integrated into a 500 MHz NMR instrument.

**Scheme 1:**
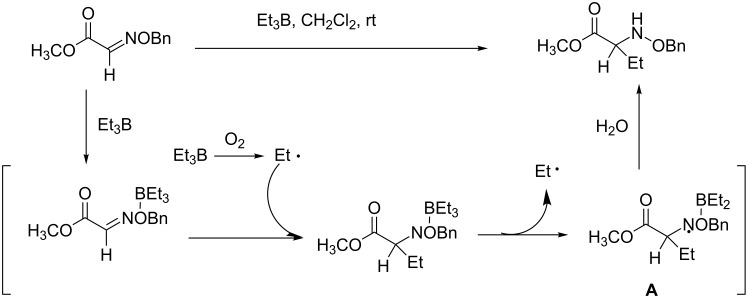
Proposed reaction mechanism.

The system was used to elucidate the mechanism of the radical addition to an oxime ether with triethylborane (Scheme 1). The use of the NMR micro flow cell permitted the detection of intermediate A, which is unstable but is crucial for the elucidation of the reaction mechanism.

The authors consider that this system has several advantages over other methods: (i) detection of short-lived intermediates is possible, (ii) two-step chemical reactions can be observed, (iii) reaction conditions can be examined very easily by real-time monitoring and (iv) integration of small amounts of products and intermediates would be possible [45].

**Figure 9:**
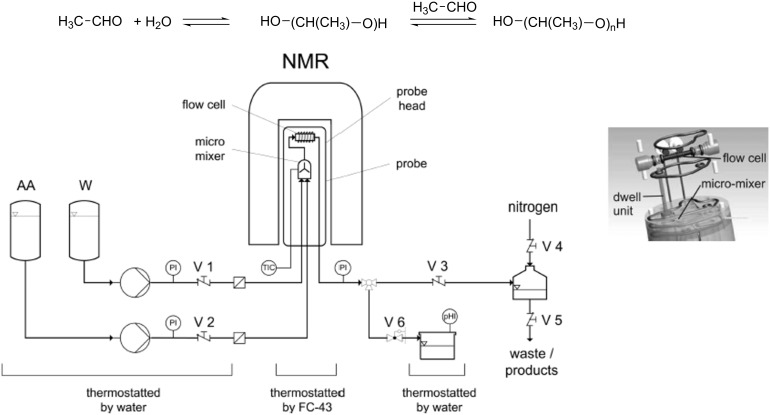
Flowsheet of the experimental setup used to study the reaction kinetics of the oligomer formation in mixtures of acetaldehyde (AA) and water (A) (Flow rates V_AA_ 254 μL min^−1^ and V_W_ 748 μL min^−1^). Reproduced with permission from reference [46]. Copyright 2014 The American Chemical Society.

Harbou et al. [46] performed a kinetic study on the multicomponent reaction of acetaldehyde and water to produce poly(oxymethylmethylene)glycols. They used a new microreactor probe head that combined online flow ^1^H NMR spectroscopy (400 MHz) using microreactor technology (Figure 9).

The microreactor NMR probe head was operated in stopped-flow. Under these conditions, the NMR flow cell is quickly filled with the reacting mixture of the desired overall composition because of the high flow rates used. The flow is then stopped and the NMR flow cell is used as a batch reactor in which the reaction is monitored online. The outlet line of the NMR probe head is connected to a vessel, which is pressurized with nitrogen to apply a back-pressure and thus adjusts the system pressure. In this way a new kinetic model could be developed for this reaction taking into consideration a wide range of temperatures and pH values. The results obtained extend the knowledge of the reaction kinetics for this industrially important system.

**Figure 10:**
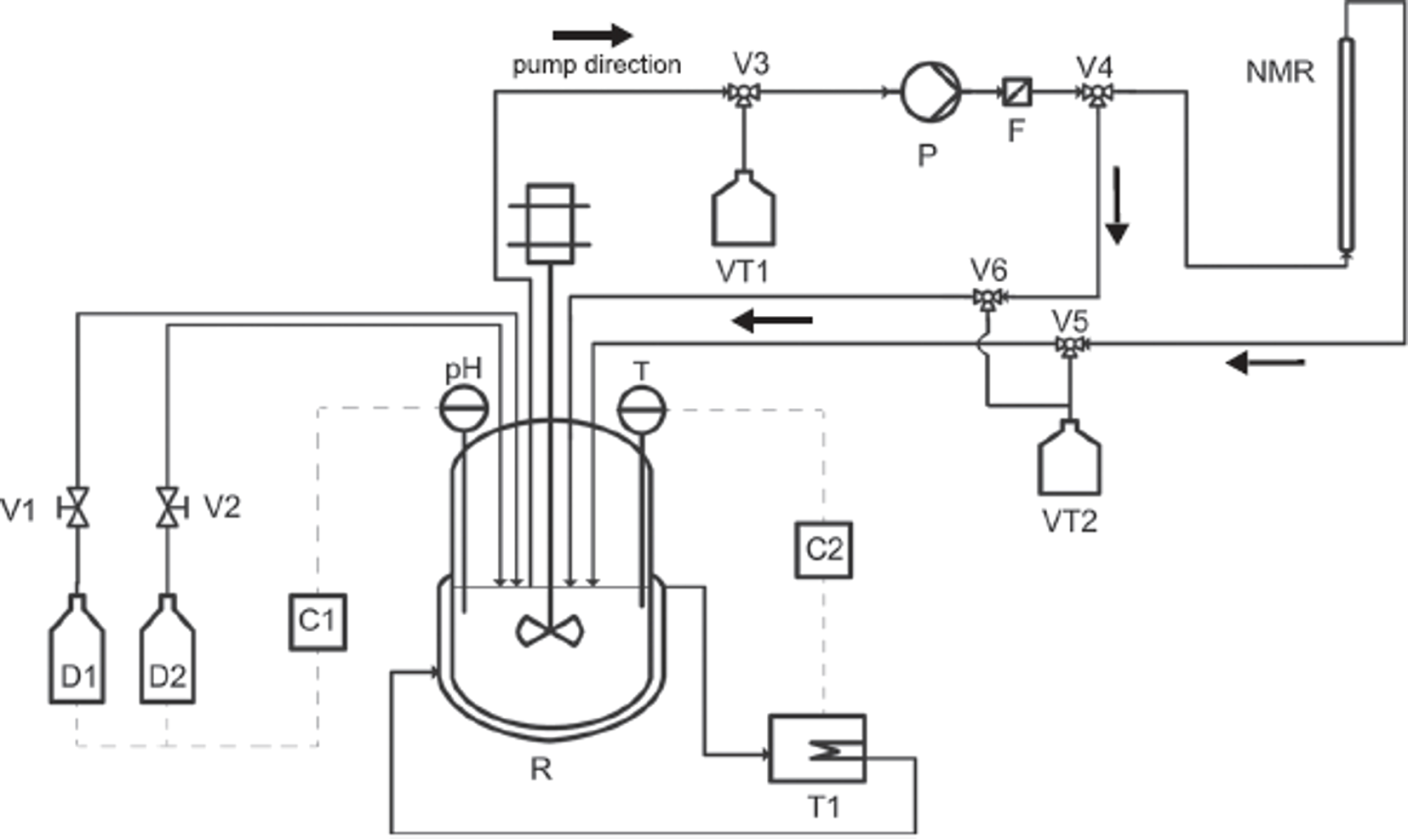
Design of the experimental setup used to combine on-line NMR spectroscopy and a batch reactor. Reproduced with permission from reference [47]. Copyright 2015 John Wiley and Sons.

Similarly, Steinhof et al. [47] studied the equilibria and kinetics of the reaction of 1,3-dimethylurea with formaldehyde, which is a model for the industrially relevant urea–formaldehyde system. The reaction was performed in a batch reactor and the analysis was carried out using a commercial NMR flow probe (Figure 10).

**Figure 11:**
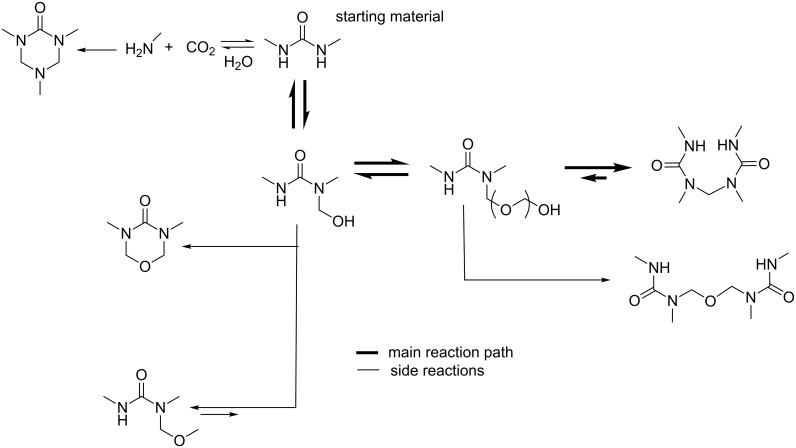
Reaction system 1,3-dimethylurea/formaldehyde. Main reaction pathway and side reactions [47].

The design represented in Figure 10 allows the regulation of the molar ratio of reagents for the kinetic experiments (urea/formaldehyde from 1:1 to 4:1) as well as the temperature and pH, which were constantly measured. The reaction mixture flowed to the NMR instrument (400 MHz) by way of a pump and, before entering the NMR flow probe, the sample was filtered and the volume selected with a split valve. In order to estimate the equilibrium time the authors performed dilutions using a micromixer prior to the NMR probe. In this way, it was possible to elucidate the reaction kinetics of the reaction system, including the main reaction pathways and also the side reactions, and to detect several intermediates including the formation of an ether bridge (Figure 11).

**Figure 12:**
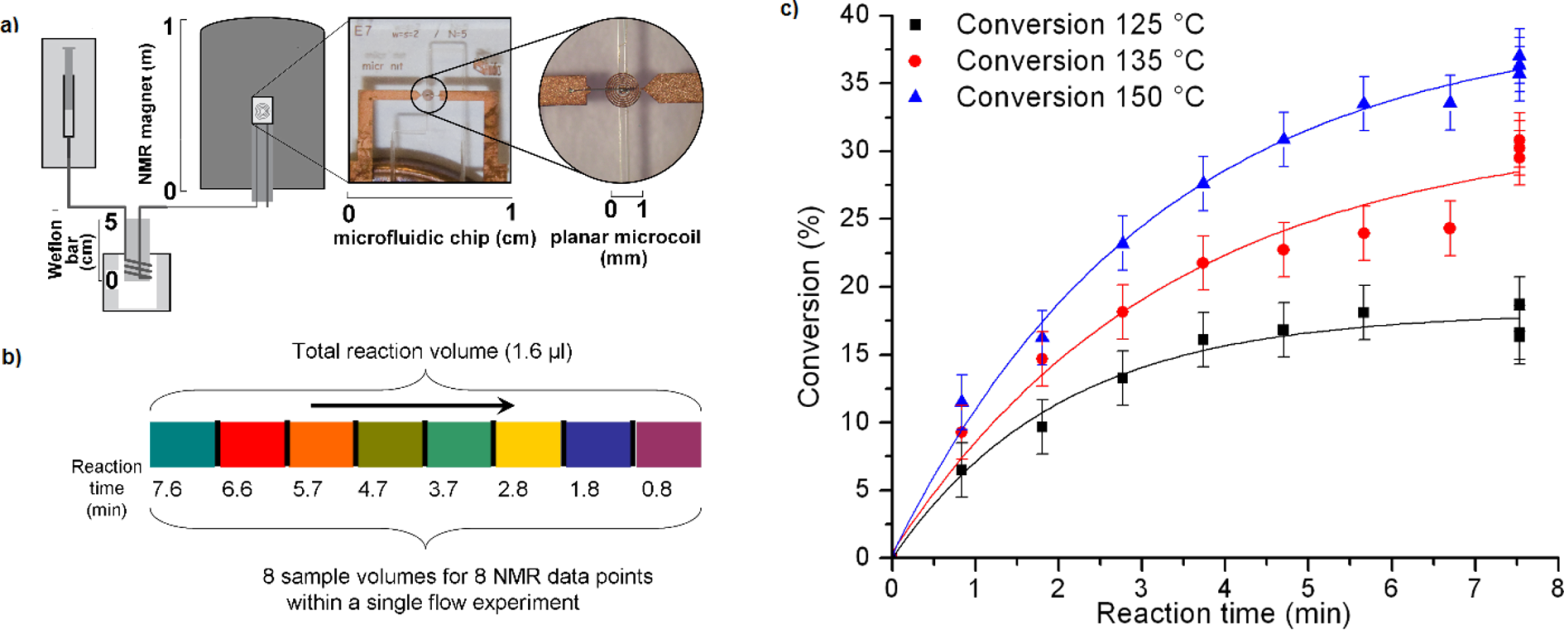
(a) Experimental setup for the reaction. (b) Reaction samples analyzed independently by NMR. (c) Plot of conversion vs time at different temperatures determined by NMR. All of the data points for a certain temperature were collected from a single flow experiment. Reproduced with permission from reference [48]. Copyright 2010 The Royal Society of Chemistry.

Gomez et al. [48] reported the first contribution that combines microstructured NMR probes with microliter continuous-flow microwave-assisted organic reactions. A microfluidic NMR chip with a planar microcoil and a detection volume of 6 nL was used for detection (Figure 12a). The specially designed microwave reactor has a small cavity in which a Weflon^TM^ (15% carbon filled PTFE) bar is introduced to ensure almost instantaneous heating. A fused silica capillary is wrapped around the Weflon^TM^ bar to ensure efficient heat transfer and the total reaction volume was 2 μL. The minimal capillary thickness also permits rapid cooling of the reaction prior to NMR analysis in a 300 MHz NMR instrument.

Considering that the detection volume is smaller than the reaction volume (Figure 12b), different fractions of the initial reaction volume can be analyzed independently. In this way, it is possible to analyze reaction volumes submitted to different irradiation times in the same on-flow experiment.

As a model reaction, the cycloaddition of 2,5-dimethylfuran with dimethyl acetylenedicarboxylate in toluene was studied. With this system the authors could optimize the reaction conditions in a rapid manner with the consumption of very small amounts of solvent and reagents (Figure 12c). It is interesting to note that this set-up worked with standard, non-deuterated solvents.

This result showed how the synergistic interaction of microwave irradiation as the energy source and the rapid reaction characterization available with NMR and flow techniques can be used for rapid optimization in a single experiment in short time and with very small solvent volumes.

Determination of the kinetic parameters for a reaction usually requires the measurement of the initial reaction rate for different initial substrate concentrations and temperatures, and fitting of the data to the corresponding reaction rate law. Overall this is a very time-consuming process. However, the use of flow-NMR techniques leads to a marked reduction in the time required for kinetic analysis. In this respect, Gomez et al. [49] reported an efficient flow system to determine kinetic information in a single experiment by taking advantage of the ability of the NMR chip, again a planar microcoil, to analyze very small volumes.

Bearing in mind once again that the detection volume is much smaller than the reaction volume, it is possible to extract information at the onset and during the steady state of the reaction, and to analyze the data to determine the kinetic parameters in a single non-isothermal on-flow experiment of 10 minutes and with a total volume of less than 50 μL.

**Figure 13:**
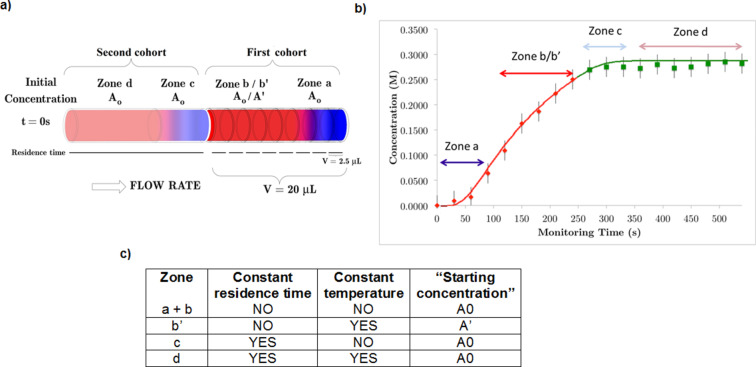
(a) Schematics of two microreactor cohorts of sample fractions. (b) Reaction product concentration (M) versus monitoring time (s) for the synthesis of 5-methyl-3-phenylisoxazole in methanol at an overall flow rate of 5 μL/min (initial concentration, 0.35 M). Every data point corresponds to a 2.5 μL fraction. (c) Table with differences between the zones (a + b, b', c, d) in residence time, temperature, and concentration. Flow-NMR analysis were performed in a 400 MHz instrument. Reproduced with permission from reference [49]. Copyright 2015 The American Chemical Society.

The first cohort consists of sample volumes ranging from monitoring time zero to *t*_R_ and the second cohort spans from *t*_R_ to 2*t*_R_. For the first cohort, the time spent in the microreactor is equal to the monitoring time *t*. The second cohort of sample fractions always spends time *t*_R_ in the microreactor (Figure 13a).

In zone a, every data point corresponds to a different temperature (Figure 13b). Zone b' shows a constant temperature value (different to zone a). Zone c includes the first data points for samples that entered the microreactor before temperature stabilization, each with a different temperature value. Finally, zone d includes the data points that experienced the constant temperature of 393 K for 4 minutes (but with a difference in ‘starting’ concentration from that of zone b').

Fitting these data, i.e., temperature, temperature gradients, starting concentration and residence times, against a reaction conversion model enables the reaction order, rate constant, Arrhenius parameters, pre-exponential factor, and activation energy values to be determined in a rapid manner from one single flow experiment.

The two latter examples reported by Gomez et al. [48–49] illustrate the advantages of combining microprobes with flow techniques. The capabilities of the microcoil of analysing very small sample volumes enable the division of the reactor volume in different portions of different experimental conditions, allowing a fast collection of experimental data and therefore, a fast optimization of reaction conditions and determination of kinetic parameters. On the other hand, some limitations and problems are encountered when combining microcoils with flow techniques. The usual limitations of working on flow NMR (i.e., clogging, bubbles, precipitation and dirty flow cells among others) are present at this small scale [28].

**Figure 14:**
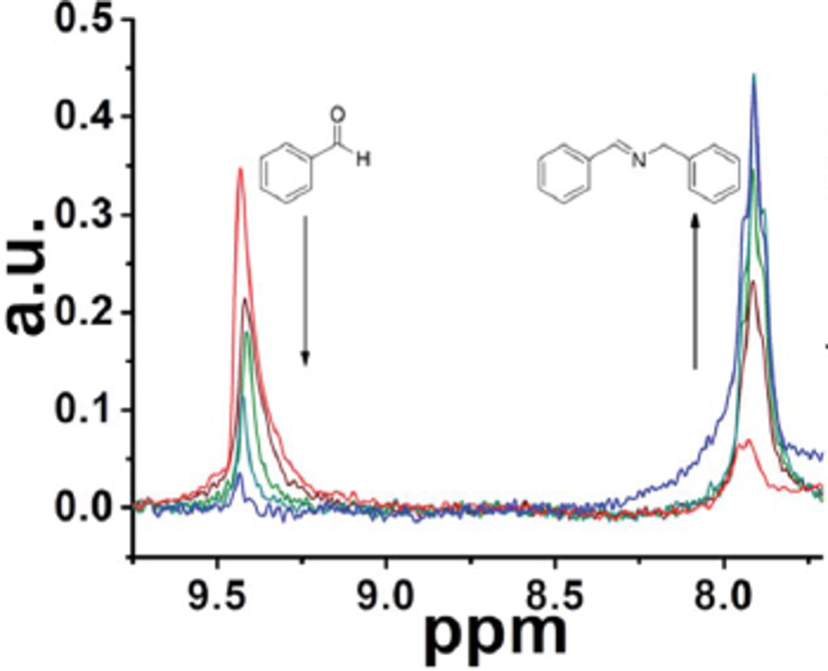
NMR analysis of the reaction of benzaldehyde (2 M in CH_3_CN) and benzylamine (2 M in CH_3_CN) (1:1), residence time, 30 min. Reproduced with permission from reference [50]. Copyright 2015 The Royal Society of Chemistry.

**Figure 15:**
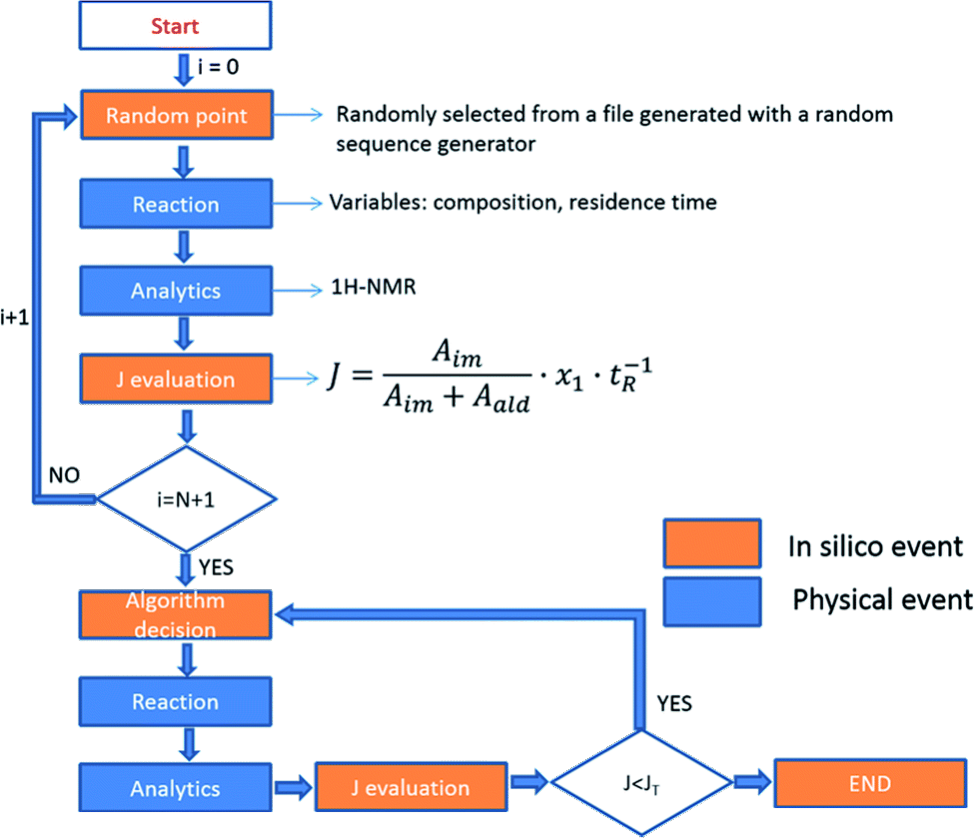
Flow diagram showing the self-optimizing reactor system. Reproduced with permission from reference [50]. Copyright 2015 The Royal Society of Chemistry.

Finally, Cronin et al. [50] described a synthetic platform that incorporates a flow reactor, an in-line benchtop NMR instrument (Spinsolve from Magritek) to monitor the organic reactions, and a control system to analyze NMR (via Labview software) data and optimize the reaction conditions. They performed a range of reactions including imine formation (Figure 14), electrophilic fluorinations and Diels–Alder reactions. This system was employed to perform kinetic studies, in-line structural characterization including DEPT spectra, 2D-NMR spectroscopy, ^19^F NMR spectroscopy and monitoring of the stereoselectivity in Diels–Alder reactions and self-optimization of flow conditions using a modified version of the Nelder–Mead algorithm. For the NMR integral data for each experiment, the algorithm (Figure 15) selects the composition and residence time for each experiment.

This study showed the potential of the combined use of flow-chemistry, real-time on-line analysis, especially by flow-NMR, and design of experiments (DOE) for the characterization and self-optimization of chemical reactions.

## Conclusion

Real-time analysis of a reaction is one of the key principles of green chemistry [51] for pollution prevention. However, on-line and in-line analysis together with the use of flow chemistry and the appropriate software for analysis, determination of the kinetic and thermodynamic parameters and for process optimization, are a key for a new type of chemistry in the 21^st^ century.

In this regard, the use of NMR spectroscopy is probably the most interesting technique of choice. Although NMR spectroscopy lacks the high sensitivity of other analytical techniques such as MS, IR, and UV–vis, it is possibly the most powerful method for structural determination and it provides an excellent platform for analysis and characterization of the reaction product.

Besides the low sensitivity of flow NMR spectroscopy some other limitations can be found. They are specific of each technique or to its combination. These limitations include:

- Clogging of the capillary tubing by precipitation of the sample, that produces a mechanical blockage and is increasingly important as the diameter of the capillary is reduced.
- Formation of bubbles it is always a problem but especially if they get into the flow NMR cell since they can distort the NMR lineshape.
- Pressure produced when using gases may produce bubbles and a reduction of the sensitivity of the NMR instrument.
- In flow reaction, a laminar flow should be assured (Re < 2000) avoiding a turbulent flow (Re > 3000). The NMR coil require a uniform magnetic susceptibility in the whole sample that cannot be assured with a turbulent flow. This problem may occur also if mixing of the components is not perfect or even when using mixtures of deuterated and non-deuterated solvents, since they have different magnetic susceptibilities.

All these limitations may affect the reproducibility and the accuracy of the quantitative analysis of the reaction, especially if mixing is not perfect, the analyzed sample may be not representative of the whole reaction.

Finally, this is an interdisciplinary field with implications in chemistry, physics, engineering and mathematics and with many possibilities of development and innovation. Further developments in microchip technology, microcoils (higher sensitivity, broadband and 2D NMR applications [52]) and improved sensitivity for benchtop NMR instruments, together with the development of new and improved software for product analysis and reaction optimization, will extend and popularize the application of these methodologies.
